# Behaviour change interventions to reduce second-hand smoke exposure at home in pregnant women – a systematic review and intervention appraisal

**DOI:** 10.1186/s12884-017-1562-7

**Published:** 2017-11-14

**Authors:** Mukesh Dherani, Syeda Nosheen Zehra, Cath Jackson, Veena Satyanaryana, Rumana Huque, Prabha Chandra, Atif Rahman, Kamran Siddiqi

**Affiliations:** 1 Department of Public Health and Policy, Institute of Psychology, Health and Society, University of Liverpool, Waterhouse Building B, Liverpool, L69 3GL UK; 2 Lancashire Care NHS Foundation Trust, Preston, UK; 3 Department of Health Sciences, University of York, York, UK; 4 Department of Clinical Psychology, National Institute of Mental Health and Neuro Sciences (NIMHANS), Bengaluru, India; 5 Department of Economics, Dhaka University, Dhaka, Bangladesh; 6 Department of Psychiatry, Institute of Psychology, Health and Society, University of Liverpool, Liverpool, UK

**Keywords:** Second-hand smoke, Behaviour change, Pregnancy health

## Abstract

**Background:**

Second-hand smoke (SHS) exposure during pregnancy is associated with poor pregnancy and foetal outcomes. Theory-based behaviour change interventions (BCI) have been used successfully to change smoking related behaviours and offer the potential to reduce exposure of SHS in pregnant women. Systematic reviews conducted so far do not evaluate the generalisability and scalability of interventions. The objectives of this review were to (1) report the BCIs for reduction in home exposure to SHS for pregnant women; and (2) critically appraise intervention-reporting, generalisability, feasibility and scalability of the BCIs employed.

**Methods:**

Standard methods following PRISMA guidelines were employed. Eight databases were searched from 2000 to 2015 in English. The studies included used BCIs on pregnant women to reduce their home SHS exposure by targeting husbands/partners. The Workgroup for Intervention Development and Evaluation Research (WIDER) guidelines were used to assess intervention reporting. Generalisability, feasibility and scalability were assessed against criteria described by Bonell and Milat.

**Results:**

Of 3479 papers identified, six studies met the inclusion criteria. These studies found that BCIs led to increased knowledge about SHS harms, reduction or husbands quitting smoking, and increased susceptibility and change in level of actions to reduce SHS at home. Two studies reported objective exposure measures, and one reported objective health outcomes. The studies partially followed WIDER guidelines for reporting, and none met all generalisability, feasibility and scalability criteria.

**Conclusions:**

There is a dearth of literature in this area and the quality of studies reviewed was moderate to low. The BCIs appear effective in reducing SHS, however, weak study methodology (self-reported exposure, lack of objective outcome assessment, short follow-up, absence of control group) preclude firm conclusion. Some components of the WIDER checklist were followed for BCI reporting, scalability and feasibility of the studies were not described. More rigorous studies using biochemical and clinical measures for exposures and health outcomes in varied study settings are required. Studies should report interventions in detail using WIDER checklist and assess them for generalisability, feasibility and scalability.

**Trial registration:**

CRD40125026666.

## Background

There is a growing body of evidence implicating second-hand smoke (SHS) exposure causally with many health outcomes such as ischaemic heart disease, lower respiratory infection, asthma, and lung cancer among non-smokers [1]. Despite a reduction in global smoking prevalence, the number of daily smokers has increased with some recent preliminary indications of an increase in smoking prevalence in men [2]. Non-smoking women, particularly pregnant women, in low-middle income countries (LMIC) are especially affected given overcrowded households and unrestricted smoking inside homes [3] leading to adverse health consequences for women and their foetuses. SHS is associated with low birth weight [4], pre-term birth [5], stillbirth [6, 7], small for gestational age [5] and congenital malformations [7]. It is estimated that more than a third of non-smoking women (35%) worldwide are exposed to SHS [1] (even during pregnancy). Indeed, the attributable risk due to SHS exposure in pregnancy could be higher than active smoking or a body mass index greater than 30 [8].

Behaviour change interventions (BCIs) have been used successfully to change smoking related behaviours. Several studies of BCIs to reduce SHS exposure have reported a reduction in SHS exposure among children [9, 10]. BCIs informed by theory were found to be particularly effective [11]. However, most studies have historically not provided enough intervention details to ascertain their theoretical basis [12]. Furthermore, it is important to identify which BCI is suited to a specific context [13]. Other limitations include poor reporting on the feasibility and scalability of such interventions [14]. In 2009, the Workshop for Intervention Development and Evaluation Research (WIDER) developed recommendations for reporting BCI interventions [14]. For generalisability, intervention description should be able to depict if it was relevant for the population and the context in which it was applied [15].

Pregnancy in every culture provides a window of opportunity to change harmful behaviours by the entire family [16] especially when the focus is the health of the foetus [16, 17]. A recently conducted systematic review [18] assessed clinical interventions, including pharmacological and psychological interventions, to reduce SHS exposure among pregnant women. The five selected studies were clinical trials which reported a significant positive effect of psychological interventions. The outcome was self-reported in three studies which were labelled as poor quality. The other two studies, whilst using objective measures, lacked details about the selection process, randomisation and adherence to the intervention. This review did not critically appraise the interventions for generalisability, feasibility and scalability. The objectives of the review presented here were to (1) report the BCIs for reduction in home exposure to SHS for pregnant women; and (2) critically appraise intervention-reporting, generalisability, feasibility and scalability of the BCIs employed.

## Methods

### Search strategy

The systematic review was guided by the PRISMA Statement for Reporting Systematic Reviews and Meta-Analyses [19]. We developed a structured search strategy using terms used for “tobacco smoke” (tobacco smoke pollution, second hand smoke, passive smoke, environmental tobacco smoke), “pregnancy” AND “intervention OR therapy OR education OR advice OR counsel” using MeSH terms or the thesaurus of the relevant databases. We limited searches to randomised trials/quasi-randomised trials and before-after studies published 2000-2015 in English.

The following databases were searched: MEDLINE; PsycINFO and CINAHL Plus through EBSCO host, Cochrane Tobacco Addiction Group Specialized Register, Database of Abstracts of Reviews of Effectiveness (DARE), Cochrane Central Register of Controlled Trials (CENTRAL) and Health Technology Assessment via Centre for Reviews and Dissemination (CRD) databases, and York CRD databases.

### Inclusion criteria

The PICO criteria were applied: (1) Population: Men attempting to change their smoking behaviours where their pregnant wife/partner is the agent of change. (2) Intervention: BCIs to reduce SHS exposure at home. (3) Comparison: no intervention or usual care. (4) Outcome: self-reported or objectively assessed (nicotine/cotinine/CO levels or clinical measures) SHS exposure of the pregnant woman at home; smoking behaviour of the man, or awareness/knowledge of the risks of SHS. Type of study design: randomised controlled trial (RCT), quasi-randomised trial or before and after studies.

### Exclusion criteria

(1) Population: Studies where children or other family members were either the target population or agents of change. (2) Intervention: Public health/community based interventions such as mass media campaigns, health policy/legislation, pharmacotherapy and complementary therapy interventions. (3) Comparison: No comparison group. (4) Outcome: no SHS outcomes.

The initial searches were conducted by one reviewer (MD). All records were imported into an Endnote database and duplicates were removed. Records were independently screened by two reviewers (MD, SZ) using the title and abstract. Relevant articles were flagged in the database. Next, the selected articles were assessed independently by both reviewers for full text review. Any discrepancies arising during the whole process were discussed and resolved between the two reviewers. A senior reviewer (AR) was available to resolve any unsettled disagreements but such occasion did not arise.

### Data extraction and quality appraisal

To address objective one, the two reviewers extracted and appraised each study using a tool adapted from a previous systematic review [20] for experimental studies. Both reviewers extracted all studies independently, the data were compared, and merged in a table. When discrepancies occurred, they were resolved by consulting the original paper.

To address objective two, the four WIDER criteria [14] were applied to each study. These criteria are: detailed description of intervention, clarification of assumed change process and design principles, access to intervention manuals/protocols, and detailed description of active control conditions. We also adapted a framework for assessing generalisability by Bonell et al. [15]. This framework comprises assessment of population acceptability, feasible delivery, local needs assessment and coverage. For scalability assessment, criteria described by Milat et al. [21] were used. These criteria, in addition to generalisability, assess size and reach, effectiveness of intervention and the context in which the intervention is delivered.

## Results

**Fig. 1:**
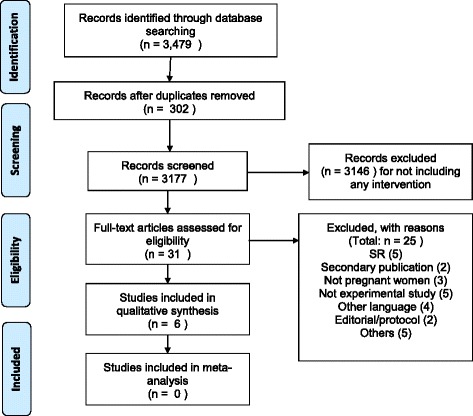
Flow diagram of included and excluded studies (PRISMA flow diagram)

The PRISMA flow diagram showing the process of study selection and reasons for excluding studies is presented in Fig. 1. The search of eight databases generated 3479 citations. After removing duplicates, sifting through titles and abstracts and removing studies that did not include an intervention, 31 publications were shortlisted for full review. Six studies were included in the final review. These are summarised in Table 1.

**Table 1:** Summary of included studies

Author (year)	Study Design and Setting	Participants	Intervention	Outcomes	Findings
El-Mohandes (2010) [22]	RCT USA	African American women at 6 prenatal care sites *I* = 335; *C* = 356	Integrated Behavioural Intervention using role play and skills practice to build negotiation skills with partner and other smoking family members and to enhance knowledge of SHS harm.	Self-reported environmental tobacco smoke exposure (ETSE), birth weight and gestational age at delivery Sub group with <20 ng/ml saliva cotinine used to represent median number with no. cigarettes smoked	Logistic regression analysis: ETSE OR 0.5 (0.35,0.71); <20 ng/ml: 0.57(0.38,0.84). LBW: *I* = 9.5%, *C* = 13.5%, *p* = 0.11; VLBW: *I* = 0.4%, *C* = 3.1%, *p* = 0.02; Pre-term birth: *I* = 11.6%, *C* = 13.5%, *p* = 0.49; Very pre-term: *I* = 1.4%, *C* = 5.6%, *p* = 0.01
Karatay (2010) [26]	Before-after study Turkey	Educated smoking pregnant women selected from ANC. *N* = 45; 38 completed	Motivational interviews based on TTM. Eight weekly visits at home with data on baseline smoking habits, raising awareness, motivation to quilt, asking all smokers not to smoke at home and evaluation of self-efficacy score.	Self-reported smoking and reduction in SHS exposure	Reduction in SHS 86.8% at first visit to 47.7% at final visit (*p* < 0.05) Measured CO and urine cotinine to verify quitting smoking but did not report if SHS reduction had any impact on these levels
Huang (2013) [24]	RCT Taiwan	Pregnant women from Urban Taiwan *I* = 65; *C* = 70	Intervention based on TTM. DVD informing about effects of SHS and strategies to avoid SHS; a booklet about the stages of change, quizzes, and exercises to reinforce the information; accessory tools such as stickers, bibs, door hangers with no smoking signs.	Stages of change i) pre-contemplation ii) contemplation/preparation iii) action/maintenance Determinants of Change i) Knowledge ii) Experiential process iii) Behavioural process Decisional Balance i) Pros ii) Cons Self-efficacy	*I* vs *C* Stages of change i) 3 (4.6%) vs 8 (11.4%) ii) 4 (6.2%) vs 12 (17.1%) iii) 58 (89.2%) vs 50(71.4%) Determinants of Change i) 15.04 SD 0.18 vs 12.46 SD 0.24 ii) 44.32 SD 0.43 vs 40.39 SD 0.51* (*p* = 0.025) iii) 38.86 SD 0.74 vs 31.83 SD 0.78 Decisional Balance i)19.27 SD 0.18 vs 18.27 SD 0.21 ii) 12.02 SD 0.51 vs 13.23 SD 0.46 Self-efficacy 16.28 SD 0.8 vs 13.29 SD 0.43 *denotes statistically significant
Kazemi (2012) [23]	RCT Iran	Pregnant women recruited from 10 health centres. 91/130 completed study; *I* = 47; *C* = 44	5 sessions with 4-week interval of education package informed by HBM. This comprised a pictorial booklet and face to face verbal session.	Perceived susceptibility Perceived severity Perceived benefits of avoiding SHS Perceived barriers to avoiding SHS Weekly ETSE exposure defined as mean number of cigarettes smoked close to pregnant woman each week by husband	Scores at final visit Perceived susceptibility *I* 17.93 SD2.23; *C* 16.29 SD 3.27 Perceived severity *I* 17.85 SD2.24; *C* 16.83 SD 2.76 Perceived benefits *I* 22.8 SD2.1; *C* 21.14 SD 2.94 Perceived barriers *I* 6.57 SD1.75; *C* 6.93 SD 1.47 Weekly ETSE: *I* 12.28 SD 15.1 *C* 25.39 SD 13.2 F-stat 8.68, *p* < 0.0001 for diff b/w groups on t-test; mean ETSE exposure difference at baseline and last week in *I* and *C*: −19.49
Loke and Lam (2005) [25]	RCT China	758 Literate pregnant women attending ANC	Intervention informed by the Theory of Reasoned Action. Standardised advice from obstetrician and an education booklet which described simple strategies helping husbands to quit smoking.	Attempt to quit in past 7 days Change in number of cigarettes per day in last month Quit smoking completely in last 7 days; quit for last 30 days or more Attempted and actual quitting. Post-intervention questionnaire was administered around 36 weeks of pregnancy.	Number of quit attempts None: *I* 266 (70%) vs *C* 294 (78%) ≥1: *I* 114 (30%) vs *C* 84 (22%); *p* = 0.02. Changes in number of cigarettes smoked *I* : 151 (39.7%) vs *C* 67 (17.7%) No change *I* 193 (50.8%) vs *C* 267 (70.7%) Increase *I* 36 (9.5) vs *C* 44 (11.6%) (*p* < 0.0001) Quit smoking in last 7 days *I* 32 (8.4%) vs *C* 18 (4.8%) (*p* = 0.04) Quit smoking for last 30 days or more: *I* 23(6.1%) vs *C* 16 (4.2%) (*p* = 0.26)
Lee (2008) [17]	Before-after study China	Non-smoking pregnant women with husband smokers recruited from antenatal clinics of 3 hospitals for 6 focus groups; *N* = 55 128 women recruited to pilot study.	Intervention informed by HBM with reference to Social Cognitive Theory. Advice from the doctor (noted in the clinical records for clinicians to reinforce the message), hot telephone line for counselling and advice delivered bi-weekly over the telephone by the researcher. Round up meeting with all to share their experiences and a resource booklet.	Knowledge about harmful components of SHS Disease due to SHS Harm to pregnancy Dislike SHS Assertive action against husband's smoking Assertive action against family member smoking	Change from before to after Knowledge of harmful components 32% to 92% (*p* < 0.01) Knowledge of disease 19.5% to 74.2% (*p* < 0.01) Knowledge of harm 38% to 73% (*p* < 0.01) Dislike SHS 51% to 83% (*p* < 0.01) Assertive action against husband 92% to 98% (*p* < 0.05) Assertive action against family member 56% to 87% (*p* < 0.01)

Four studies were RCTs [22–25] and two were before-and-after studies without a control group [17, 26]. Two studies were from high income countries [22, 24]. The sample size ranged from 45 to 758. Only one study included non-smoking pregnant women [26]. All studies recruited the study participants from antenatal clinics (ANC). One study included non-pregnant women attending paediatric clinics (only data for pregnant women were included in this review) [24]. Two studies used the Health Belief Model (HBM) [17, 23], two used the Transtheoretical Model (TTM) [24, 26], one used an Integrated Behavioural Intervention [22] and one used the Theory of Reasoned Action (TRA) [25]. The interventions were in a variety of formats ranging from advice from doctors, a telephone hot-line, one-to-one consultation, motivational interviews, video, role play, information booklet and accessory articles such bibs and hangers with reminder messages about the harms of SHS. In one study, the intervention was delivered at home [26], the others were delivered in hospital clinics. Two studies [22, 26] reported objective measures for SHS exposure, but only El-Mohandes [22] used these data in the analyses. Karatay et al. [26] used this information to verify self-reported quitting. One study assessed the impact of reduction in SHS on pregnancy outcomes [22].

### Study intervention

The RCT by El-Mohandes [22] in the USA, was carried out in six prenatal care sites where 691 (intervention = 335; control = 356) non-smoking African American pregnant women < 28 weeks gestation were recruited. The integrated behavioural intervention was delivered to the women by trained psychologists or social workers over eight sessions, of approximately 35 min [27]. It included role plays and skills practice to develop negotiation skills and to enhance knowledge about the harmful effects of SHS.

In the before-and-after study carried out in central Turkey [26], 38 of 45 participants completed the study. All participants were educated pregnant women who smoked and 87% also reported being exposed to SHS at home. The motivational interviewing intervention, a component of the TTM, was delivered during eight home visits. The fourth visit included a meeting with all smokers at home to discuss the importance of not smoking indoors.

Huang et al. [24] carried out a RCT in urban Taiwan. Sixty-five pregnant women were in the intervention group and 70 in the control group. The intervention was based on the TTM. A video was shown to participants followed by education material to reinforce the video message. The research staff explained to women the need to focus on the material corresponding to their TTM stage. Two telephone follow-ups, one occurring two weeks after the intervention, and then another a week later, were carried out by a research nurse.

Kazemi et al. [23] recruited 130 pregnant women, of which 91 (47 intervention and 44 control) completed the trial. The intervention was based on the HBM and aimed to increase the sense of susceptibility to SHS and to improve understanding of benefits of reducing SHS exposure. It was delivered by trained midwives in five one-to-one sessions with the first session lasting for 15-20 min and remaining sessions lasting 5-10 min. The intervention also included a booklet containing simple terms and pictures to impart knowledge.

Loke and Lam [25] recruited 758 literate pregnant women to a RCT (380 in the intervention and 378 in the control arm). The intervention was informed by the TRA and delivered by an obstetrician during the ANC visits with an educational booklet suggesting strategies for the women’s husbands to stop smoking at home. During the next follow-up visit, the women were asked about the steps taken to avoid SHS exposure, and a reminder by the obstetrician was also given.

Lee [17] carried out a mixed-method study with a qualitative component to develop an intervention which was then piloted in a before-and-after study. They recruited 55 non-smoking pregnant women for two rounds of focus group discussions to develop an intervention based on the HBM with reference to Social Cognitive Theory, and 128 women to pilot test the intervention. The intervention comprised advice by the obstetrician, an information booklet, access to support via a telephone hotline and bi-weekly follow-up reinforcement over the telephone by the research team.

### Appraisal of intervention reporting

**Fig. 2:**
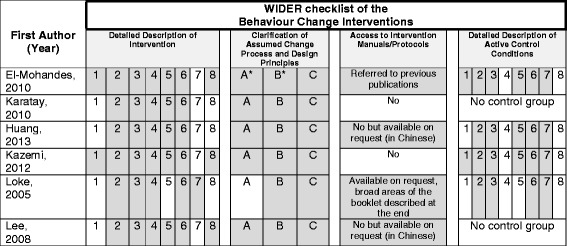
WIDER Checklist for intervention appraisal. Legend. *Grey* = recommendation met. *White* = recommendation not met. *Reference to articles are mentioned by same author on intervention development. Detailed description of intervention/active control conditions: 1) Characteristics of those delivering the intervention/control condition 2) Characteristics of the recipients 3) Setting 4) Mode of delivery 5) Intensity 6) Duration 7) Adherence/ fidelity to delivery protocols 8) Detailed description of the intervention/control content. Clarification of Assumed Change Process and Design Principles: A) Intervention development described B) Change techniques employed in intervention identified and described C) Causal processes targeted by change techniques identified and described. This Figure has been reproduced with permission from the authors

Figure 2 presents the WIDER checklist for intervention reporting. The majority of the studies fulfilled the criteria to some extent. Three items: recipient characteristics, setting and mode of delivery were reported in all six studies. All provided information about the theory behind the intervention. Only one study mentioned the availability of a detailed protocol upon request [25] and two provided information in Chinese if contacted [17, 24]. No study reported any information on the contents of the intervention received by the control group.

The study by El-Mohandes [22] referred to the detailed process of development of the intervention in a previous publication [28] based on behaviour change literature. Their Integrated Behavioural intervention model was informed by the TTM, social ecological and cognitive behavioural treatment models. The authors did not describe how adherence to the delivery protocol was monitored in the intervention group or explain how contamination with the control group, selected from the same clinics, was avoided. They referred to the previous publication for a detailed protocol [28].

Karatay et al. [26], described the details of the intervention development based on the TTM using four behavioural techniques: the emphatic approach, developing discrepancy, solving resistance and supporting self-efficacy. Additionally, they offered a suggested mechanism for the proposed change in behaviour. They did not describe who delivered the intervention. A description of participants who were literate women was given but the recruitment process was not described. There was no control group for the study. Similarly, information on adherence to the protocol was not provided. A detailed protocol was not available.

Huang et al. [24] provided a detailed description of the inventory used for each stage of the TTM. They described who delivered the intervention (research staff and nurses), however they did not provide detailed characteristics or describe any training provided to them to carry out these tasks. As a study limitation, they mentioned possible contamination of the control group. A detailed protocol in Chinese can be accessed by contacting the authors.

Kazemi et al. [23] used the HBM to develop their intervention. A description of study participants and study setting was provided. Trained midwives provided the intervention but details of their training were not described; nor was there any description of measures taken to evaluate adherence to the study protocol. The detailed protocol is not accessible.

Loke and Lam [25] referred to a theory-based intervention as the basis for their intervention. They did not provide any information about the development of the intervention; nor did they provide information on the intensity of the intervention apart from describing follow-ups after 3-5 months, presumably, indicating the intervention duration. They asked study participants to complete a follow-up slip to demonstrate if the health advice (intervention) was given by the physicians. They mentioned access to a detailed protocol and provided salient features of the resource booklet in the publication.

The pilot study in China by Lee [17] provided a detailed intervention development process and characteristics of the participants. However, they neither described the characteristics of those delivering the intervention nor assess adherence to the protocol delivery. The study did not include a control group. The detailed protocol in Chinese is accessible by contacting the author.

### Generalisability, feasibility and scalability

Table 2 summarises the assessment of studies for generalisability, feasibility and scalability, using the above-mentioned tools [15, 21]. None of the six studies achieved all three.

**Table 2:** Assessment of generalisability, feasibility and scalability

Study	Generalisability	Feasibility	Scalability
El-Mohandes (2010) [22]	No	Yes	No
Karatay (2010) [26]	No	No	No
Huang (2013) [24]	No	Yes	No
Kazemi (2012) [23]	Yes	Yes	No
Loke (2005) [21]	No	Yes	Yes
Lee (2008) [17]	No	Yes	No

The El-Mohandes study [22] was carried out in a Black minority population, and given that the cultural and behavioural patterns and needs vary from population to population and ethnic groups, even within a country or region, it is unlikely that such interventions may be generalisable to the wider population. The intervention was delivered by Master’s level graduates. Feasibility is uncertain in situations where trained professionals are not available. The effect size, one of the scalability criteria, is fairly large for two outcomes (see Table 1), but information on scalability of such specialist-led approaches was lacking. Additionally, the researchers did not consider the readiness of the health system that would implement the intervention should this be scaled up.

Karatay et al. [26], used purposive sampling to select participants, all of whom were literate. Literacy may be an important factor limiting the generalisability as well as feasibility in settings where female literacy rates are low. The intervention was delivered during eight home visits making it difficult to adopt due to resource constraints faced by most health systems. During the intervention phase researchers also found some women regressed from action to an earlier intention phase of behaviour change. Whilst this was not a large number of participants, it will impact on the effect size and may limit the scalability.

Huang et al. [24] randomly selected a study population from an urban setting and the study results may be generalizable to urban Taiwanese population. The intervention appears to be acceptable but certain elements like arranging a DVD player and providing a tailored explanation to an individual woman about her stage of change may be resource intensive, thus limiting the feasibility. The level of training required for an interventionist to understand the model and explain to the pregnant women in a language they understand may also hamper the feasibility and scalability. The effect size was high and statistically significant. Additionally, as acknowledged by the authors, a longer follow-up was required to assess if the intervention impact was sustainable.

In the RCT in Iran [23] a random selection of participants from a number of hospitals makes the results more generalizable. It seems feasible as midwives delivered the intervention whilst women attended routine ANC appointments, so potentially having the opportunity to deliver the intervention within these appointments. Although in reality, busy ANC schedules may make it difficult for the midwives to deliver the intervention. Also, it is not clear if the opinion of the midwives regarding the intervention delivery were taken into consideration.

The intervention using an obstetrician to provide health advice in RCT in China [25] is feasible but the participants were recruited from one major hospital only, and were literate. Hence, generalisability may be questionable. The advice from doctors/health professionals may be scalable but to incorporate this in health system requires policy change as the advice on SHS exposure reduction is not routinely provided. The short-term effects of the intervention, number of attempts to quit smoking and quitting smoking in last seven days, were statistically significant (*p* < 0.05). Longer term quitting for 30 days was high in the intervention group but not statistically significant.

The pilot study by Lee [17] was the only study that incorporated population views in developing the intervention through focus group discussions. The researcher acknowledged the lack of generalisability due to convenience sample selection. The intervention component of advice from a doctor is feasible but other components like a telephone hot line may make it difficult to scale-up. The effect size of the intervention was high.

## Discussion

To our knowledge this is the first systematic review that appraises BCIs applied to the pregnant women to target change their husband/partners’ smoking behaviours using the WIDER checklist [14]. Moreover, this review also evaluates BCIs for generalisability, feasibility and scalability. Despite high prevalence of SHS exposure and strong evidence of the health risks, only a small number of intervention studies were available [18].

### BCIs for reduction in home exposure to SHS for pregnant women

In the six studies selected for this review, the BCIs administered showed a low to moderate success in achieving the selected outcomes. BCIs are generally considered as effective tools for changing harmful behaviours [9–11] but unless robust methodology and a systematic approach are employed their impact may lack internal and external validity [29] or may even be ineffective. In this review, few studies reported sample size calculations [25, 26] which make it difficult to discern if the effect size was real. Both of the before-and-after studies lacked a control group [17, 26].

Outcomes are more reliable if they are objective. Most studies in this review used self-reported smoking behaviours and knowledge as outcomes. Without an objective measure, it is not possible to know if changes in knowledge and husbands/partners’ smoking behaviour actually reduce SHS exposure and improve pregnancy outcomes. For example, SHS exposure from other family members and visitors may persist. Only one study [22] reported objective outcome measures, such as cotinine levels in urine and saliva or health outcomes. The review by Tong et al. also reported a paucity of literature with objectively measure outcomes and recommended using the biochemical measures to reduce the biases [18]. However, whilst biochemical markers are more robust measure of recent smoking behaviour, they are expensive to assess. More research is required to evaluate the feasibility and effectiveness of the biochemical markers.

All the studies had a short follow-up after intervention, so the longer-term impact on health outcomes such as child cognitive development was not assessed. Similarly, it was not possible to predict if outcomes such as increases in knowledge translated into a reduction in SHS exposure. A trial in China reported that a reduction in smoking habits at three months reverted to no change at 12 months. The authors argued that the alteration in motives such as improvement in child health may have led to the relapse [30]. We suggest that future studies should have a longer follow-up evaluating health outcomes as well as the sustainability of intervention impact.

### BCI-reporting and their generalisability, feasibility and scalability

The second objective of this review was to critically appraise intervention-reporting, and the generalisability, feasibility and scalability of the BCIs employed. To improve science, it is deemed necessary to identify what worked and how it worked, in order to replicate and improve interventions in the local context [14].

The studies reviewed only partially met the WIDER criteria. Most of the interventions comprised multiple components that were insufficiently described. Without these details, it is not possible to fully understand the intervention or elucidate the inter-relationship between the different components or their effect on outcomes. Four studies offered access to the study protocol (two in Chinese). No studies reported a detailed description of the control intervention apart from stating that it was a standard/routine care. Describing this would help to understand on going practice and may explain any behavioural change in the control group during the study course.

With recent advances in the development and application of a taxonomy of behaviour change techniques [12], it is now possible to map BCIs to evidence-based theories. However, researchers should offer a comprehensive description of the BCIs in their papers to help readers and reviewers understand their theoretical basis. To improve such reporting, the scientific reporting standards and journal editors should expect authors to include not only a comprehensive description of BCIs, but also a logic model linking these to behaviour change theories. Research funders could also ask for such details when assessing research bids on BCIs.

No studies met all three generalisability, feasibility and scalability criteria [15, 21]. None described if their results were relevant to other populations. This is important for policy and practice decision making [31]. The information about the effectiveness of the intervention at the study site should be supplemented with information about the context [15]. Additionally, intervention content and delivery should be acceptable to the population under study. None of the studies reported data on refusal to participate or acceptability of the intervention. Indeed, apart from one study [17] no studies took their target population views into account. A further notable limitation of the studies was a lack of information regarding intervention fidelity. Only one study [25] took measures to assess if doctors gave the advice to the pregnant women as described in the protocol. An inbuilt process that gauges the adherence to the protocol is required to evaluate if the process was applied without any bias in a uniform and standard manner.

## Conclusion

Behaviour change related to smoking is an important area of research, especially when it impacts on a woman’s pregnancy and the health of her foetus. This review indicates that while there have been several studies using different BCIs for reducing home exposure to SHS among pregnant women, they suffer from major limitations and are not easily adaptable in different countries and different settings. More rigorous studies using biochemical and clinical measures for exposure and health outcomes in varied study settings are required. Studies should report BCIs in detail using WIDER checklist and assess them for generalisability, feasibility and scalability.
